# Cell Surface Glycosylation Is Required for Efficient Mating of *Haloferax volcanii*

**DOI:** 10.3389/fmicb.2017.01253

**Published:** 2017-07-05

**Authors:** Yarden Shalev, Israela Turgeman-Grott, Adi Tamir, Jerry Eichler, Uri Gophna

**Affiliations:** ^1^School of Molecular and Cell Biology and Biotechnology, George S. Wise Faculty of Life Sciences, Tel Aviv UniversityTel Aviv, Israel; ^2^Department of Life Sciences, Ben-Gurion University of the NegevBeersheva, Israel

**Keywords:** archaea, glycosylation, lateral gene transfer, mating, surface layer

## Abstract

Halophilic archaea use a fusion-based mating system for lateral gene transfer across cells, yet the molecular mechanisms involved remain unknown. Previous work implied that cell fusion involves cell–cell recognition since fusion occurs more efficiently between cells from the same species. Long believed to be restricted only to Eukarya, it is now known that cells of all three domains of life perform N-glycosylation, the covalent attachment of glycans to select target asparagine residues in proteins, and that this post-translational modification is common for archaeal cell surface proteins. Here, we show that differences in glycosylation of the *Haloferax volcanii* surface-layer glycoprotein, brought about either by changing medium salinity or by knocking out key glycosylation genes, reduced mating success. Thus, different glycosylation patterns are likely to underlie mating preference in halophilic archaea, contributing to speciation processes.

## Introduction

The intriguing phenomenon of lateral gene transfer mediated by cell fusion in halophilic archaea (class Halobacteria, phylum Euryarchaeota), also known as mating, has been recognized since the 1980s (Mevarech and Werczberger, 1985; Rosenshine et al., 1989; Rosenshine and Mevarech, 1991; Tchelet and Mevarech, 1993). More recent work has shown that such fusion events can also mediate reciprocal homologous recombination events between different *Haloferax* species that span hundreds of thousands of base pairs (Naor et al., 2012). Such cell fusion events are not, however, restricted to halophilic archaea. Two species of *Thermococcus* (class Thermococci, phylum Euryarchaeota), for which no genetic tools exist, were also shown to fuse in the presence of a DNA-interchelating dye (Kuwabara et al., 2005, 2007), indicating a possible connection between DNA exchange and cell fusion that may be more conserved in archaea than previously appreciated.

Cell fusion in the genus *Haloferax* has been shown to be more efficient within than between species (Naor et al., 2012), implying that a specific cell–cell recognition process is involved in this semi-specificity. Given that the most dominant molecule on the surface of *Haloferax* cells is the surface-layer (S-layer) glycoprotein, the sole component of the S-layer surrounding the cell (Sumper et al., 1990), it is likely this protein, and potentially its covalently linked glycans, plays some role in the cell fusion process. In *H. volcanii*, the S-layer glycoprotein is subjected to both N- and O-glycosylation (Sumper et al., 1990; Eichler et al., 2013). Indeed, S-layer glycoprotein N-glycosylation occurs at multiple sites that can be affected by environmental cues (Guan et al., 2012; Kaminski et al., 2013a). Since protein-linked sugars are known to mediate cell–cell interactions, as well as cell–matrix interactions, in many eukaryotic cells (for review see Varki and Lowe, 2009), N-glycosylation of haloarchaeal S-layer glycoproteins may play a role in mediating cell–cell recognition within a species and promote initiation of the fusion process.

With this in mind, we examined how differences in *H. volcanii* surface glycosylation influenced cell fusion and showed that environmental and genetic perturbations to this process were able to dramatically affect fusion efficiency. This suggests that surface glycosylation may play a role in cellular recognition and within-species mating preferences in halophilic archaea, thereby affecting gene exchange and speciation processes.

## Materials and Methods

### Mating Protocol

As described previously (Naor et al., 2012), cultures of mating partner strains were grown to an OD_600nm_ of 2.0, and 1 ml aliquots were drawn from each and applied to a 0.2 μm filter connected to a vacuum to eliminate excess medium. The filter was then placed on a Petri dish containing a rich medium (Hv-YPC medium + thymidine, see below) for 24 h at 42°C. The cells were washed, re-suspended in Hv-Ca broth, washed twice more in the same medium, and plated on selective media. Plates were incubated at 42°C until colonies were large enough to be counted.

### Measuring Cell Fusion Efficiencies

Mating efficiency was calculated as the number of mating product CFUs on the selective plates divided by the average number of CFUs of each parental strain (for strain genotypes see **Table 1**). Mating efficiencies of *H. volcanii* incubated at different salt concentrations were calculated using the auxotrophic strains H53 (Δ*pyrE*,Δ*trpA*) and H729 (Δ*hdrB*), selecting for uracil and thymidine as chromosomal markers. Mating efficiencies of strains with defects in N-glycosylation of the S-layer glycoprotein were assessed using episomal plasmid markers. *H. volcanii* Δ*aglB* cells (based on H53 and lacking the oligosaccharyltransferase AglB) were described previously (Abu-Qarn et al., 2007). The Δ*aglB*Δ*agl1*5 strain was created by deleting the *agl15* gene from Δ*aglB* cells. Gene knockouts were performed according to the protocols described in (Lam and Doolittle, 1989; Bitan-Banin et al., 2003). Mating partner strains were transformed using the PEG method (Mevarech and Werczberger, 1985), such that one strain was transformed to carry the plasmid pWL-nov and the other plasmid pWL-102. Selection on plates containing the resistance markers coded by these plasmids, i.e., novobiocin (pWLnov) and mevinolin (pWL102), was then performed.

**Table 1:** Archaeal strains and plasmids used in this work.

Source/Reference	Description	Strain/plasmid
T. Allers	*H. volcanii*Δ*hdrB*	H729
T. Allers	*H. volcanii*Δ*pyrE2*Δ*trpA*	H53
Abu-Qarn et al., 2007		*H. volcanii* Δ*aglB*
This study		*H. volcanii* Δ*aglB*Δ*agl15*
Abu-Qarn et al., 2007		*H. volcanii* Δ*aglD*
Ortenberg et al., 2000	Resistance to novobiocin	pWL-nov
Lam and Doolittle, 1989	Resistance to mevinolin	pWL-102


### Culture Conditions

*Haloferax volcanii* cells were routinely grown as described (Allers et al., 2010).

### Cryo-TEM Analysis of RSO Membrane Vesicles

Right-side out (RSO) membrane vesicles were prepared from WT glycosylation and Δ*aglB*Δ*agl15* strain cells and examined by cryo-TEM as described previously (Tamir and Eichler, 2017).

### Glycan Detection

The glycan moiety of the S-layer glycoprotein was glycostained by periodic acid-Schiff’s reagent, as previously described (Dubray and Bezard, 1982).

### ImageJ Analysis

SDS–PAGE band intensity was analyzed using ImageJ (Schneider et al., 2012).

## Results

### Mating in *Haloferax volcanii* Is More Efficient at Higher Salt Concentrations

**FIGURE 1:**
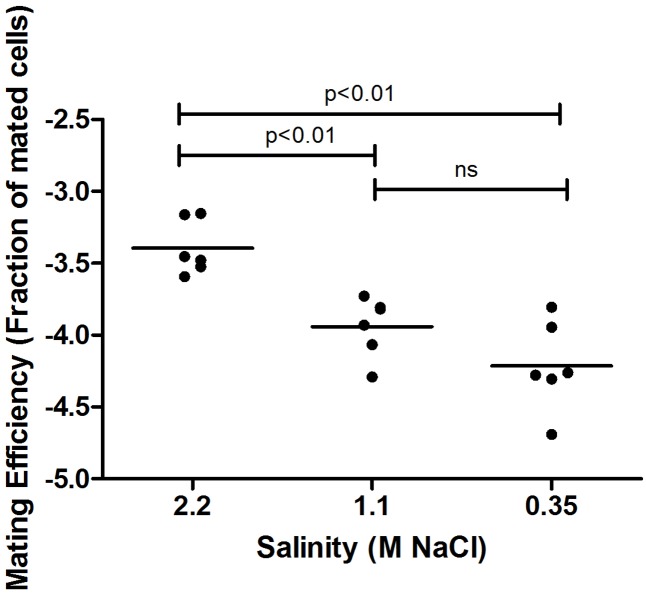
Mating efficiency of *Haloferax volcanii* in different salinities. A comparison of cell fusion efficiencies between strains cultivated at different NaCl concentrations. The experiment was performed by allowing 24 h for mating and selecting for chromosomal markers (see Materials and Methods). In each column, the horizontal line corresponds to the mean. In addition, different samples were compared by a two-sided Mann–Whitney *U* test. The *p*-value is provided in each case.

**FIGURE 2:**
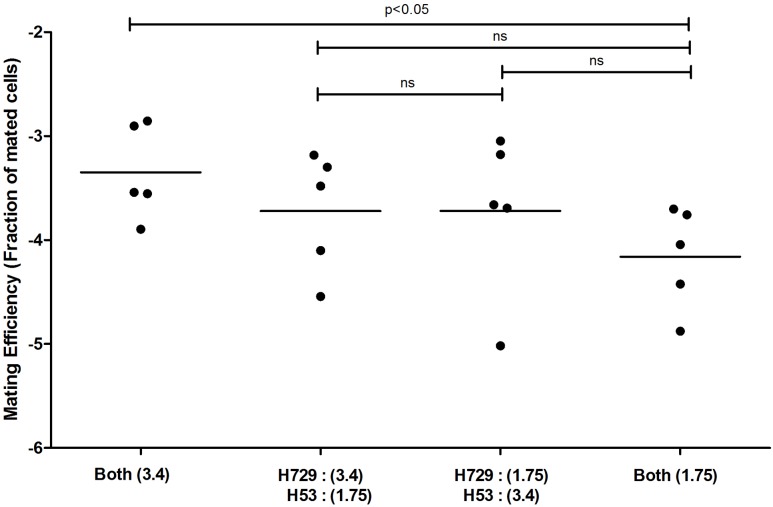
Mating efficiency in *Haloferax volcanii* of parental strains exposed to different salinities. A comparison of cell fusion efficiencies between strains in which each parent was cultivated separately at different NaCl concentrations. The experiment was performed by allowing 24 h for mating and selecting for markers located on the main chromosome (see Materials and Methods). The salinity (M NaCl) in each case is given in parenthesis. In each column, the horizontal line corresponds to the mean. In addition, different samples were compared by a two-sided Mann–Whitney *U* test. The *p*-value is provided in each case.

Haloarchaea are defined by their ability to grow in hypersaline solutions. Yet, although *Haloferax* species require high salt (2.2 M NaCl) concentrations for optimal growth, they are able to replicate in salinities as low as 0.7 M NaCl and survive prolonged exposure to seawater (Jantzer et al., 2011). Moreover, variation in environmental salt concentrations was previously shown to result in differential processing of N-glycosylation sites in the *H. volcanii* S-layer glycoprotein by glycans containing distinct sugar compositions (Guan et al., 2012). We thus hypothesized that such changes could affect mating efficiency in this species. As such, cultures grown overnight under standard conditions were incubated in fresh medium containing different NaCl concentrations, defined as optimal, medium and low salinity media (2.2, 1.1, and 0.35 M NaCl, respectively), prior to mating. In each mating assay, two parental strains, H729 (Δ*hdrB*) and H53 (Δ*pyrE*Δ*trpA*), that had been incubated at the same salinity were mated on membrane filters, as previously described (Naor et al., 2012). Mated colonies were selected based on their ability to grow on casamino acid-containing medium lacking uracil and thymidine. Such selection revealed a positive association between mating efficiency and increasing salinity. On average, higher mating efficiency was obtained when cells where incubated in 2.2 M NaCl, as compared to those incubated at 1.1 M NaCl (*p* < 0.01, two-tailed Mann–Whitney test), with the lowest efficiency being observed in 0.35 M NaCl (**Figure 1**). When the H53 and H729 parental strains grown in 3.4 or 1.75 M NaCl surroundings were allowed to mate, it was sufficient to have only one mating partner initially incubated at the higher salinity for effective mating at a level closer in efficiency to that obtained when both strains were incubated at the optimal salt concentration of 2.2 M NaCl (**Figure 2**, *p* < 0.05, two-tailed Mann–Whitney test, for mating between parents from 3.4 M NaCl and parents from 1.75 M NaCl; the difference in efficiency between cases where one parental strain was incubated in 3.4 M NaCl vs. both strains incubated in 1.75 M NaCl, mating did not reach statistical significance). This implies that at least some aspect of the fusion process in *H. volcanii* is asymmetrical in nature, and that cells that are mating-proficient can display high mating efficiency with non-optimally grown partners.

### Interfering with S-Layer Glycoprotein N-Glycosylation Leads to Decreased Mating Efficiency

As mentioned above, *H. volcanii* modulates the sites and composition of N-linked glycans decorating the S-layer glycoprotein in response to changes in environmental salinity (Guan et al., 2012). Since alterations in salt concentrations also brought about changes in mating frequency, it was hypothesized that modulation of N-linked glycans decorating the S-layer glycoprotein could explain the observed variance in mating efficiency. Specifically, the possibility that differential S-layer glycoprotein N-glycosylation could affect cell–cell recognition and, hence, mating frequency, was considered.

**FIGURE 3:**
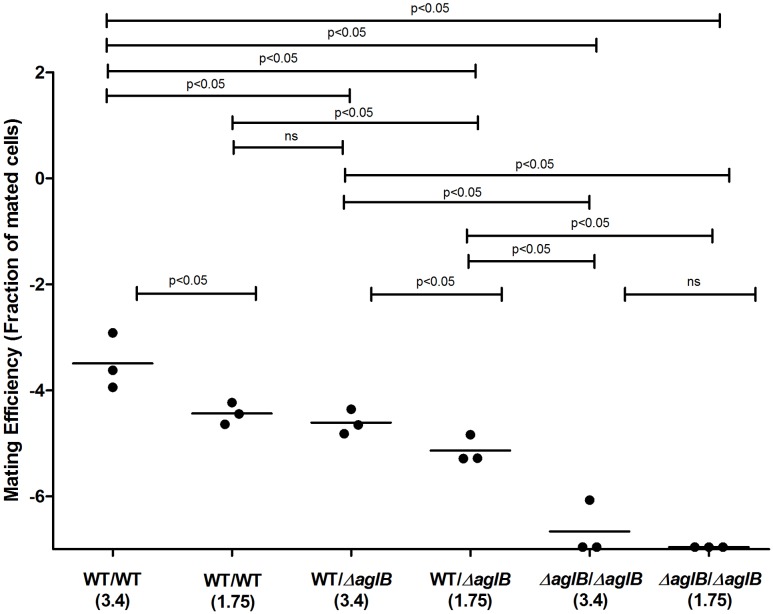
Mating efficiencies of *H. volcanii* with WT glycosylation and Δ*aglB* strains. Comparison of cell fusion efficiencies between WT glycosylation strains and strains with S-layer glycoproteins lacking the N-linked pentasaccharide. The salinity (M NaCl) in each case is given in parenthesis. In each column, the horizontal line corresponds to the mean. In addition, different samples were compared by a one-sided Mann–Whitney *U* test. The *p*-value is provided in each case.

Previous efforts have defined the *H. volcanii* pathways responsible for the assembly of the pentasaccharide N-linked to at least four S-layer glycoprotein Asn residues and of the tetrasaccharide attached to a distinct Asn residue when cells are grown in only 1.75 M NaCl-containing medium (Eichler et al., 2013; Kaminski et al., 2013a). To test whether deficient S-layer glycoprotein N-glycosylation reduces mating efficiency, *H. volcanii* strain deleted of genes encoding central components of each of these pathways were used. In cells lacking *aglB*, the oligosaccharyltransferase responsible for delivering the first four sugars of the N-linked pentasaccharide attached to S-layer glycoprotein Asn-13, -83, -274 and -279 from a common dolichol phosphate carrier is absent, such that these residues are not glycosylated (Abu-Qarn et al., 2007; Kandiba et al., 2016). The Δ*aglB* strain was crossed with a strain (H729) that shows wild type glycosylation in mating efficiency assays, selecting for mating products based on plasmid markers. The mating efficiency experiments were performed at two different NaCl concentrations (i.e., in the presence of 1.75 or 3.4 M NaCl), conditions in which two different patterns of N-linked glycosylation of the S-layer glycoprotein occur (Guan et al., 2012). Mating efficiencies in WT glycosylation strain cells containing AglB were higher when the cells were incubated at 3.4 M NaCl, as compared to those in the less saline 1.75 M NaCl-containing medium. The same trend was observed when mating of the WT and Δ*aglB* strain was assayed. WT/Δ*aglB* strain mating was, on average, less efficient than WT/WT (*p* < 0.05 in either salinity, one-tailed Mann–Whitney test) mating performed at same salt concentrations (**Figure 3**). The most profound effect on mating efficiency was observed when crossing Δ*aglB* cells with Δ*aglB* cells in either 1.75 or 3.4 M NaCl-containing medium. Here, mating efficiency was remarkably low (*p* < 0.05, one-tailed Mann–Whitney test, in either salinity, compared to WT/WT and WT/Δ*aglB* mating efficiencies), with only few, and often no mating products being observed after 140–160 h of incubation. This indicates that in *H. volcanii*, a direct link exists between S-layer glycoprotein N-glycosylation and mating by fusion, with partial dominance of the WT N-glycosylation phenotype in mating.

**FIGURE 4:**
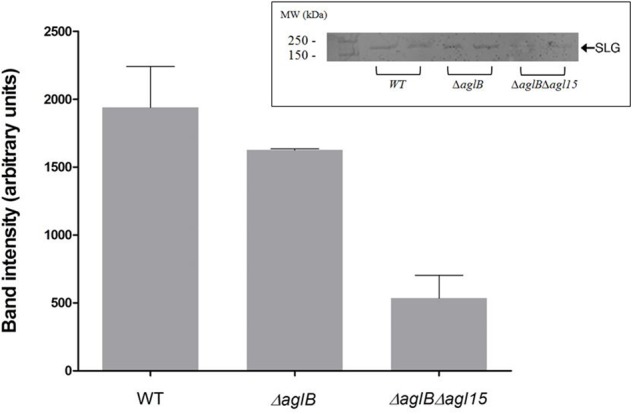
S-layer glycoprotein glycosylation. S-layer glycoproteins from a strain with WT glycosylation, Δ*aglB* cells and Δ*aglB*Δ*agl15* cells (two repeats of each strain), as revealed by SDS–PAGE and staining with periodic acid-Schiff’s reagent are shown in the inset (upper right corner). The positions of molecular weight markers are shown on the left, while the position of the S-layer glycoprotein is shown on the right. Glycostained band density was analyzed using ImageJ (see Materials and Methods). The error bar represents the standard deviation.

**FIGURE 5:**
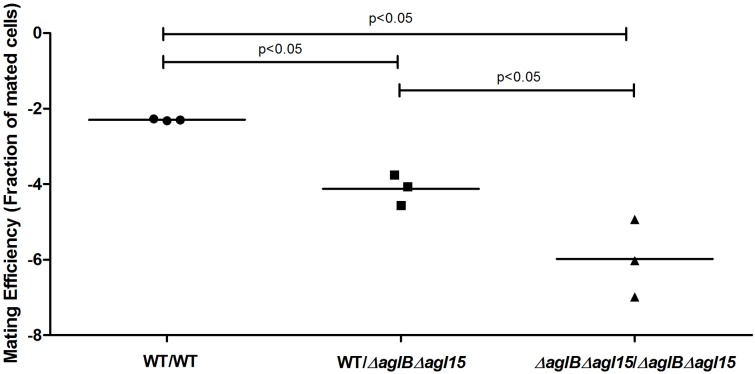
Mating efficiencies of *H. volcanii* cells presenting WT glycosylation with Δ*aglB*Δ*agl15* cells. A comparison of cell fusion efficiencies between WT glycosylation strains and strains lacking enzymes that respectively mediate or contribute to the attachment of the pentasaccharide and the low salt tetrasaccharide to the S-layer glycoprotein. The salinity used was 3.4 M NaCl. In each column, the horizontal line corresponds to the mean. In addition, different samples were compared by a one-sided Mann–Whitney *U* test. The *p*-value is provided in each case.

While the *H. volcanii* S-layer glycoprotein Asn-13, -83, -274 and -279 positions are modified by a pentasaccharide comprising a glucose, a glucuronic acid, a galacturonic acid, a methylated glucuronic acid and a mannose when cells are grown in the presence of either 1.75 or 3.4 M NaCl, the Asn-498 position is modified by a distinct tetrasaccharide comprising a sulfated hexose, two hexoses and rhamnose in cells grown in 1.75 M NaCl-containing medium (Kaminski et al., 2013a; Kandiba et al., 2016). Furthermore, it has been shown that in cells deleted of genes involved in the assembly of the N-linked pentasaccharide grown in 3.4 M NaCl, the ‘low salt’ tetrasaccharide attached to Asn-498, normally only observed upon growth in 1.75 M NaCl, was detectable, despite the elevated surrounding salinity (Kaminski et al., 2013a). Therefore, deletion of *aglB* may lead to increased activity of the second N-glycosylation pathway, normally functional at the lower salinity, at higher salt levels, too. To, therefore, rule out the possibility that aberrant N-glycosylation, rather than reduced N-glycosylation, was responsible for the impaired mating phenotype observed using the Δ*aglB* strain, a double deletion strain lacking both *aglB* and *agl15* was generated. Agl15 is thought to participate in the translocation of dolichol phosphate bearing the ‘low salt’ tetrasaccharide across the membrane, such that deletion of the encoding gene leads to the absence of this glycan on the S-layer glycoprotein (Kaminski et al., 2013a). As expected, when the protein content of Δ*aglB*Δ*agl15* cells was separated on SDS–PAGE and glycostained using periodic acid-Schiff’s reagent, a drastic reduction in S-layer glycoprotein glycosylation was observed (**Figure 4**). These under-glycosylated cells also showed very low, or even undetectable, mating efficiency, even when grown at the higher salinity for mating (3.4 M NaCl; **Figure 5**) and incubated for about 210 h (about 3 days longer than the experiment described in **Figure 3**). It would thus seem that the dramatic decline in mating efficiency (*p* < 0.05, one-tailed Mann–Whitney test) seen with the double mutant strain is due to a lack of N-glycosylation of the S-layer glycoprotein comprising the S-layer, rather than differences in S-layer glycoprotein N-linked glycan composition.

### S-Layer Integrity Is Compromised in Membrane Vesicles Prepared from Cells Lacking *aglB* and *agl15*

**FIGURE 6:**
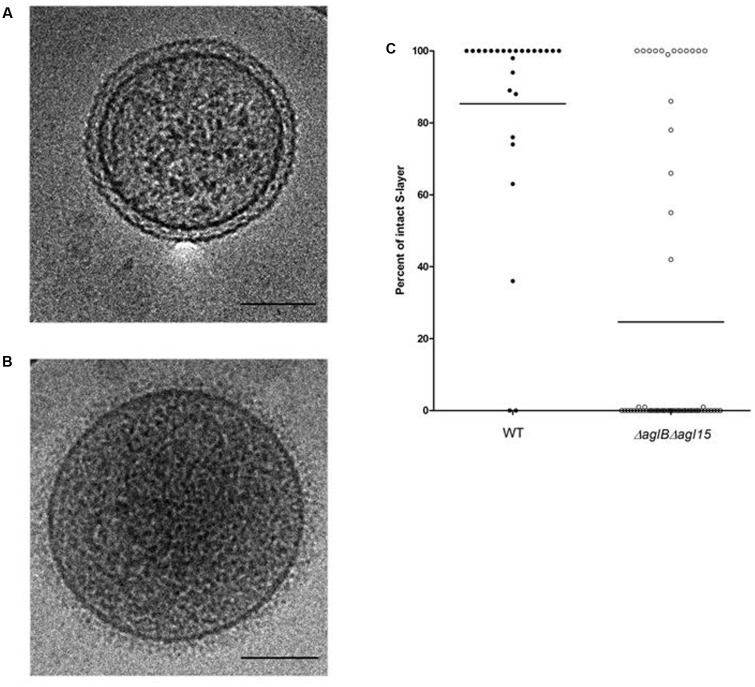
Cryo-transmission electron microscopy of right-side-out membrane vesicles. The S-layer is compromised in RSO membrane vesicles prepared from *H. volcanii* Δ*aglB*Δ*agl15*. **(A,B)** Electron micrograph of a representative RSO membrane vesicles derived from *H. volcanii* cells of the WT glycosylation strain **(A)** and the Δ*aglB*Δ*agl15* strain **(B)**. In each panel, the bar represents 100 nm. **(C)** The percentages of intact S-layer in RSO membrane vesicles prepared from cells of the WT glycosylation strain (left, full symbols; *n* = 26) and the Δ*aglB*Δ*agl15* strain (right, open symbols; *n* = 62). In each column, the average (horizontal line) and the distribution of values collected from each vesicle population are presented.

To better understand how the compromised N-glycosylation seen in the Δ*aglB*Δ*agl15* strain could lead to reduced mating, RSO membrane vesicles were prepared from WT glycosylation and Δ*aglB*Δ*agl15* strain cells and examined by cryo-transmission electron microscopy (cryo-TEM). In agreement with earlier studies (Tamir and Eichler, 2017), RSO vesicles prepared from WT glycosylation strain cells clearly showed both the plasma membrane and the surrounding concentric S-layer. The S-layer was intact, regularly ordered and equidistant from the enclosed membrane vesicle at all points (**Figure 6A**). In contrast, whereas membrane vesicles of similar dimensions as obtained from WT glycosylation strain cells were generated from the double mutant strain, in the vast majority, no surrounding S-layer was seen. Instead, a poorly ordered and more diffuse structure surrounded these vesicles (**Figure 6B**). Statistical analysis of the extent of S-layer coverage of RSO vesicles prepared from the WT glycosylation and Δ*aglB*Δ*agl15* strains confirmed these claims, with the percentage of S-layer coverage of parent strain-derived vesicles being 85.4% [±29.2% (*SD*); *n* = 26], as opposed to 24.8% (±41.4%; *n* = 62) in Δ*aglB*Δ*agl15* strain-derived vesicles. As such, it would seem that the S-layer in cells lacking AglB and Agl15 is less sturdy than in WT glycosylation cells, as reflected in how poorly this structure remained intact during the preparation of RSO vesicles from the double mutant strain.

### Minor Changes in S-Layer Glycosylation Do Not Decrease Mating Efficiency

**FIGURE 7:**
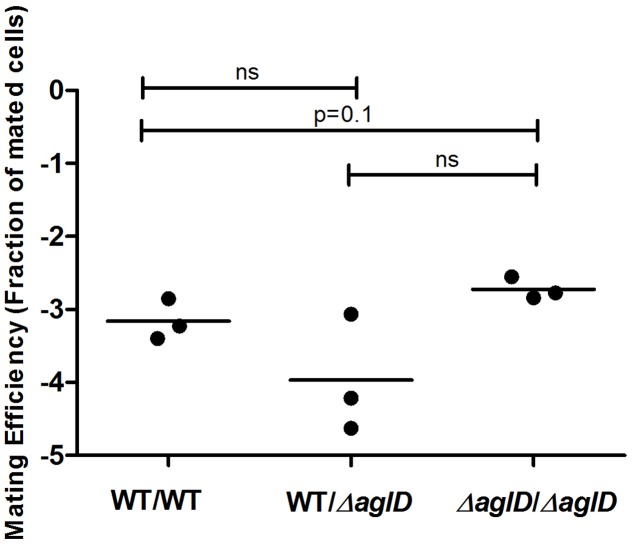
Mating efficiencies of *H. volcanii* WT and Δ*aglD* strains. A comparison of cell fusion efficiencies between WT glycosylation strains and strains lacking *aglD* that encodes an enzyme involved in adding the final sugar of the N-linked pentasaccharide decorating the S-layer glycoprotein. The salinity used was 3.4 M NaCl. In each column, the horizontal line corresponds to the mean. In addition, different samples were compared by a one-sided Mann–Whitney *U* test. The *p*-value is provided in each case.

The N-glycosylation pathway mutants generated above demonstrated drastic effects on S-layer glycoprotein glycosylation. To now test whether minor changes in N-glycosylation also perturbed mating, mating involving a *H. volcanii* strain deleted of *aglD*, encoding the glycosyltransferase that adds the final sugar of the N-linked pentasaccharide, mannose, to its own dolichol phosphate carrier (Abu-Qarn et al., 2007; Guan et al., 2010), was considered. When mating two Δ*aglD* cells, a slight increase in mating efficiency was observed, relative to the WT glycosylation strain (*p* = 0.1, two-tailed Mann–Whitney test, **Figure 7**). In contrast, a slight decrease in average mating efficiency was observed when cells of the WT glycosylation and Δ*aglD* strains were crossed, which did not reach statistical significance. This finding shows that the absence or presence of the last sugar of the N-linked pentasaccharide decorating the S-layer glycoprotein affects recognition between two *H. volcanii* cells, albeit to only a moderate extent.

## Discussion

Here, we showed that haloarchaeal fusion is affected by environmental exposure, and that higher mating efficiency is observed under high salt conditions. Given that previous work showed that mating frequently occurs in biofilms (Chimileski et al., 2014), it is likely that in their natural environment, *H. volcanii* cells fuse primarily on high-salt biofilms, such as those found on salt-covered rocks, where mating is relatively efficient.

Apart from environmental exposure, mating efficiency is also affected by the genetic capacity for S-layer glycosylation. Our results show that S-layer glycan deficiency dramatically decreased mating ability. When neither the tetrasaccaride nor the pentasaccaride were present on the S-layer glycoprotein comprising the S-layer, far fewer cell fusion events were observed. Thus, surface glycosylation is likely to be a pre-requisite for mating in *Haloferax*. However, we found that having one mating-proficient partner appears to be sufficient, since the effects of incubation of one partner in low salinity, be it an *aglB*, or a combined *aglB* and *agl15* deletion strain, on mating with the WT strain were only semi-recessive.

One explanation for the effect of loss of glycosylation on mating efficiency could be related to structural considerations (Imperiali and O’Connor, 1999). This would explain why the presence of an intact and sturdy S-layer, as seen in WT glycosylation cells, better enabled cell fusion interactions than did the N-glycosylation mutants examined, with their less hardy S-layer (**Figure 6**). In contrast to what was seen upon total loss of N-glycosylation, we observed that minor changes in N-glycosylation, such as those generated by a deletion of *aglD*, did not decrease and even slightly increased cell fusion efficiency (**Figure 7**). One reason for this could be that the methylated glucuronic acid at position four of the pentasaccharide, exposed in the absence of the final sugar (mannose) in Δ*aglD* cells (Abu-Qarn et al., 2007; Kandiba et al., 2016), mediates stronger interactions with the S-layer of neighboring cells. However, recent work showed that modification of the S-layer glycoprotein by only the first four sugars of the N-linked pentasaccharide resulted in a more protease-susceptible conformation (Tamir and Eichler, 2017) than when the same protein was modified by the complete pentasaccharide or by N-linked glycans containing three or fewer sugars. Thus, it is possible that the fifth sugar (mannose) somehow masks charges associated with other sugars in the N-linked pentasaccharide (Tamir and Eichler, 2017), thereby resulting in improved binding between cells.

The partial tolerance of mating by fusion to incompatible sugar presentation on the S-layer glycoprotein may also explain the relatively high efficiency with which different species of *Haloferax* were shown to mate (Naor and Gophna, 2013), despite differences in S-layer glycan composition (Cohen-Rosenzweig et al., 2014). The semi-dominant effect of glycosylation on mating strongly suggests a mechanism in which a protein or lipid ligand in one cell interacts with a glycosylated receptor on its partner (or vice versa) in the cell–cell recognition step required to initiate fusion (Naor and Gophna, 2013). Interacting with a specific surface sugar could thus contribute to the preference for fusion between cells of the same species (con-specific fusion), as previously observed when comparing fusion efficiencies between *H. volcanii* and *H. mediterranei* (Naor et al., 2012). Since glycosylation clusters have been frequently horizontally transferred in halophilic archaea (Kaminski et al., 2013b), the changes in S-layer glycosylation that such acquired clusters can bring could change mating preferences and result in rapid lineage diversification leading to sympatric speciation.

## Author Contributions

IT-G, UG, and YS conceived the study, IT-G and YS designed, generated and characterized mutant strains and plasmids, IT-G, YS, and UG designed experiments, IT-G, YS, and AT performed experiments and analyzed data, YS, IT-G, UG, and JE wrote the manuscript. All authors read and commented on the manuscript.

## Conflict of Interest Statement

The authors declare that the research was conducted in the absence of any commercial or financial relationships that could be construed as a potential conflict of interest.
